# Iowa State Dental Society

**Published:** 1881-02

**Authors:** 


### IOWA STATE DENTAL SOCIETY.

The volume of Transactions of the Eighteenth Annual Session, held July 13th to 15th, 1880, at Oskaloosa, has just come to hand. It is neatly gotten up, containing 89 pages. It is well filled with interesting and instructive matter, a part of which is a number of very good papers. This volume gives evidence of unmistakable progress in the dental profession of Iowa. The Register will, probably, help disseminate some of the good things in this book.

